# Effects of evenamide in a neurodevelopmental animal model of schizophrenia suggest potential sustained clinical benefit

**DOI:** 10.1192/j.eurpsy.2026.12197

**Published:** 2026-07-31

**Authors:** R. Anand, A. Turolla, G. Chinellato, F. Sansi, R. Hartman

**Affiliations:** 1 APC, St.Moritz, Switzerland; 2 Newron Pharmaceuticals SpA, Bresso, Italy; 3 NeurWrite, Morristown, United States

## Abstract

**Introduction:**

The hippocampus is regarded as the primary site of dysfunction in schizophrenia and offers a promising target for therapeutic intervention (Lodge and Grace, *TIPS* 2011). Hippocampal hyperexcitability is thought to play a key role in the pathophysiology of schizophrenia. As none of the currently available antipsychotics (APs) affect hippocampal activity, they are unlikely to alleviate cognitive dysfunctions or negative symptoms, as these are controlled by nuclei above the basal ganglia.

Evenamide, a voltage-gated sodium channels modulator, attenuated stimulated release of glutamate in an in vivo microdialysis rat model; due to its higher affinity for the inactivated state of the channels, it reduces neuronal hyperexcitability. Recently, evenamide was investigated by Uliana et al (*NPP* 2025) in the MAM (methylazoxymethanol acetate, a DNA alkylating agent) neurodevelopmental animal model of schizophrenia which revealed that evenamide exerts its action in the hippocampus.

**Objectives:**

To provide new insights into evenamide’s mechanism of action

To interpret clinical findings from phase 2/3 trials in light of the results obtained with MAM model

**Methods:**

Electrophysiological recordings in the MAM model assessed the effects of evenamide on neuronal activity in the hippocampus and ventral tegmental area (VTA). Behavioral outcomes were evaluated using the novel object recognition (cognitive function) and social approach (negative symptoms) tests.

**Results:**

MAM-treated pups as adults show features consistent with schizophrenia: loss of hippocampal parvalbumin neurons, hippocampal hyperactivity, hyperdopaminergic state, impaired cognitive function and social deficits (Lodge and Grace, *TIPS* 2011). Evenamide normalized hippocampal pyramidal neurons hyperactivity and reduced dopaminergic (DA) activity in the VTA of MAM rats; interestingly, effects persisted long after administration of evenamide despite its rapid pharmacokinetic profile. Additionally, evenamide restored impaired recognition memory and improved social interaction.

These results are in line with data from clinical trials, in which evenamide add-on treatment was associated with significant improvement of positive and also negative symptoms (PANSS subscales) in patients with inadequate response to APs (Anand et al, *Neuropharm* 2025). Moreover, persistence of effects is consistent with long-lasting benefits observed in a 1-year open-label trial in patients with TRS (Anand et al, *IJNP* 2025); long-term efficacy of evenamide compared to standard of care will be confirmed in the ongoing phase 3, 1-year, double-blind ENIGMA-TRS 1 trial.

**Conclusions:**

Evenamide’s distinct mechanism of action targeting the hippocampus differentiates it from all available APs. By modulating upstream glutamatergic dysregulation, evenamide may provide broader and more durable efficacy across various schizophrenia symptom domains.

**Disclosure of Interest:**

R. Anand Consultant of: Newron Pharmaceuticals SpA, A. Turolla Employee of: Newron Pharmaceuticals SpA, G. Chinellato Employee of: Newron Pharmaceuticals SpA, F. Sansi Employee of: Newron Pharmaceuticals SpA, R. Hartman Consultant of: Newron Pharmaceuticals SpA

